# Ketosis-prone diabetes mellitus in an obese adolescent: A case report

**DOI:** 10.1097/MD.0000000000016076

**Published:** 2019-06-21

**Authors:** Huiwen Tan, Chun Wang, Yerong Yu

**Affiliations:** a Division of Endocrinology and Metabolism, West China Hospital of Sichuan University, Chengdu; b Laboratory of Endocrinology and Metabolism, West China Hospital of Sichuan University, Chengdu, Sichuan, PR China.

**Keywords:** clamp test, classification, hyperglycemia, Ketosis-prone diabetes mellitus, remission

## Abstract

**Rationale:**

In recent years, there are more new insights into the clinical susceptibility, pathophysiological mechanism, and progression of classification and treatment of ketosis-prone diabetes mellitus (KPDM), which was once described as Idiopathic Type 1 Diabetes, Type 1B Diabetes or Flatbush Diabetes. ketosis-prone diabetes mellitus is still a heterogeneous syndrome reported in African-American or western Sub-Sahara-African, Hispanic descendant, and recently in Asian.

**Patient concerns:**

An obese 17-year-old student was admitted to a tertiary referral hospital (teaching hospital), presenting with thirst, polyuria fatigue, and a 9 kg weight loss in the preceding two weeks.

**Diagnoses:**

Physical examination showed body mass index (BMI) was 32.77 kg/m^2^, arterial blood gas revealed a pH of 7.31. Serum glucose was 27.8 mmol/L with strong positive uric ketones (++++). Hemoglobin A1c (HbA1c) was 13.6%. The glucose disposal ratio (GDR) during the steady-state of euglycemic clamp test was 5.62 mg/kg/min and M value was 2.87 mg/kg/min during hyperglycemic clamp test. Those findings were sufficient to establish a diagnosis of ketosis-prone diabetes mellitus.

**Interventions:**

This obese patient with KPDM received intensive insulin therapy and fluids infusion, and during the remainder of hospitalization his insulin requirement was approximately 1.5 U per kilogram of body weight per day. Blood glucose monitoring was rigorous until the diabetic ketoacidosis under control.

**Outcomes:**

He achieved the near-nomalglycemic remission uneventfully. At 12-month follow-up, his treatment was adjusted from insulin subcutaneous injection to oral hypoglycemic drugs.

**Lesson:**

The present study of this obese adolescent with negative auto-antibodies but unprovoked diabetic ketoacidosis and partially preserved beta cell functional reserve after the acute of diabetic ketosis suggested that he has the phenotype of “A–β^+^” KPDM. Further study of this syndrome will help illustrate the inadequacy of current classification and targeted therapies.

## 1 Introduction

The prevalence of type 2 diabetes increases in Chinese due to a rising incidence of obesity in youth and children.^[1]^ As clinicians, we have to face difficult situation in young diabetic patients with diabetic ketoacidosis. Ketosis-prone diabetes mellitus (KPDM), which was previously described as Idiopathic Type 1 Diabetes, Type 1B Diabetes or Flatbush Diabetes, are reported in in African-American, Hispanic descendant and Asian.^[2–5]^ KPDM was found be with mix characteristics of classic type 1 and type 2 diabetes.^[6]^ Here, we present a clinical observation of an adolescent with new-onset ketosis prone diabetes, which illustrates the insufficiencies of the current classifications.

## 2 Case presentation

### 2.1 Ethical review and patient consent

The present study is dealt with the patient's medical records, related laboratory tests and imaging reports. This observational study was approved by the Ethics Committee of West China Hospital of Sichuan University (No. ChiCTR-ECS-07000063). And the clinical research was implemented according to the principles expressed in the World Medical Association *Declaration of Helsinki* and the International Ethical Guidelines for Biomedical Research Involving Subjects (GIOMS, Geneva, 1993). Written informed consent was given from the patient on each occasion of diagnostic examinations and therapeutic procedures and also for the publication of this case report.

### 2.2 Case report

L.K, an obese 17-year-old student in high school, presented to the emergency center of a tertiary referral hospital with increasing thirst, frequent urination, fatigue, and a 9.0 kg weight loss in the preceding 2 weeks. Evaluation by the emergency center revealed idiopathic diabetic ketoacidosis without clinical evidence of other precipitating illnesses or stressful events. He denied the abuse of alcohol, tobacco or drugs before. No over-intake of sugar-containing foods including soft drinks. His family history was strongly positive for adult-onset diabetes. Both the patient and his mother have long-standing obesity. He had a history of borderline diastolic hypertension that had been diagnosed half a year previously and was treated with a low-salt diet. The past medical history was otherwise unremarkable.

### 2.3 Physic examination

His temperature was 36.5°C, heart rate was 117 beats per minute with weak peripheral pulses, blood pressure was 135/95 mm Hg. He was 179 cm tall and weighed 105 kg, with BMI 32.77 kg/m^2^. Physical findings were remarkable for abdominal obesity with waist circumference of 99 cm. Mild acanthosis nigricans was present on his neck and axillae. His respiratory rate was 24 breaths per minute with a Kussmaul pattern and smelled of acetone. His fingers and toes were cool, with a prolonged capillary-refill time. Examination of the lungs and heart revealed no abnormalities and the abdomen was soft, with mild, diffuse tenderness but no guarding or rebound. There was no clinical evidence of diabetic retinopathy, neuropathy or nephropathy.

### 2.4 Laboratory tests

The results of biochemistry examinations are shown in Table 1. Arterial blood gas revealed a pH of 7.31 and partial pressure of carbon dioxide of 22 and bicarbonate of 3. Serum sodium 144 mmol/L, potassium 6.9 mmol/l, bicarbonate 7 mmol/l, blood urea nitrogen 50 mg/dl, and creatinine 3.5 mg/dl. Serum glucose was 27.8 mmol/L. Hemoglobin A1c (HbA1c) was 13.6%. Serum triglyceride was 7.8 mmol/L, free fat acid concentration were 1.05 mmol/L. Serum acetone was detectable in moderate quantity. C-peptide was non-detectable. Urinalysis demonstrated a glucose concentration of more than 1000 mg per deciliter (56 mmol per liter) with strong positive ketones(++++) and negative protein. His serum amylase was in normal range. The white-cell count was 15,000 per cubic millimeter, and the hematocrit was 40%. Islet-associated autoantibody (IAA), Insulinoma associated antigen 2 (IA-2), Islet cell antibodies (ICA) and glutamate decarboxylase (GAD-65) antibody were negative. There was hepatic adipose infiltration by abdominal ultrasound.

**Table 1:**
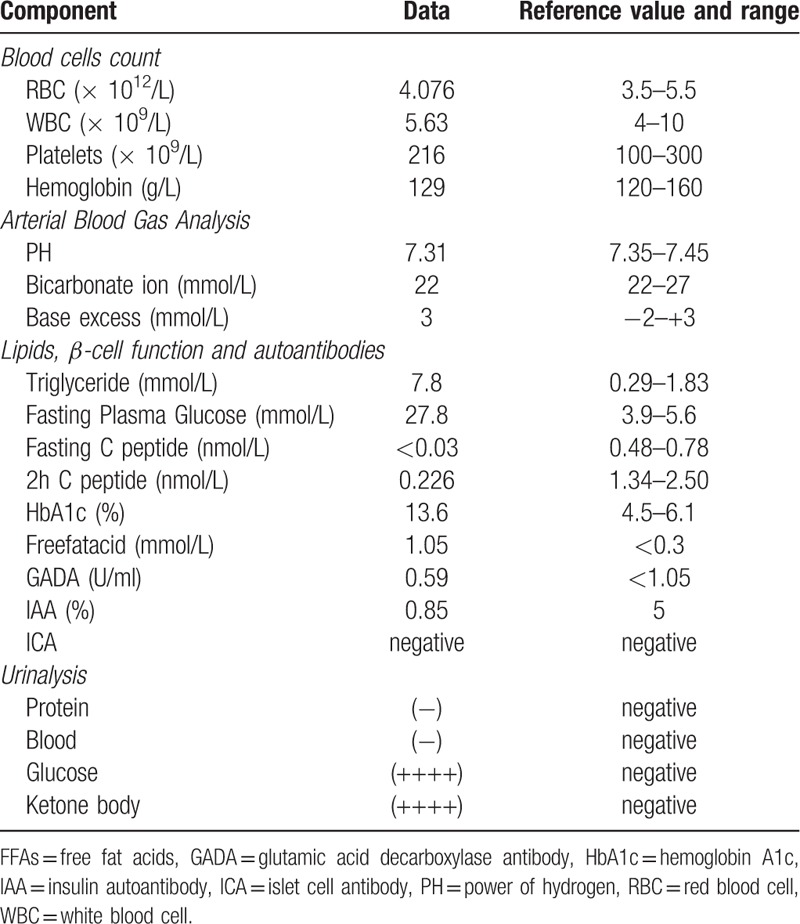
Laboratory findings of the patient with KPD at the time of admission.

He underwent euglycemic clamp test for valuation of insulin sensitivity and pancreatic beta cell function when his condition quickly stabilized in three days. The glucose disposal ratio (GDR) during the steady-state of euglycemic clamp test was 5.62 mg/kg/min and glycemic metabolism (M) value was 2.87 mg/kg/min during hyperglycemic clamp test, which means there were low insulin sensitivity and extremely deficient in insulin secretion.

### 2.5 Treatment and follow-up

The patient was admitted to the Endocrinology and Metabolism ward for treatment with intravenous fluids and insulin. He recovered uneventfully. The acidosis resolved, and during the remainder of hospitalization his insulin requirement was approximately 1.5 U per kilogram of body weight per day. At discharge he was converted to intensive subcutaneous insulin therapy with a regimen of glargine and insulin Aspart at a dose of 1.2 U per kilogram per day. Two weeks after presentation, the boy was discharged to home on a regimen of 85 units glargine before bedtime and 30 units of as part before each meal, for a total daily insulin dose of 175 units. One week after discharge, he revisited the outpatient clinic. He had regained some weight, and his post-meal glycemic control was in good condition with HbA1c of 9.8%. He was instructed to continue the insulin regimen titrated according to blood glucose values. Insulin injections were gradually discontinued after discharge and the patient was initially treated with metformin (1700 mg per day) and gliclazide extentab (30 mg per day) and subsequently with metformin (1000 mg per day). He reported near-normal glycemic remission in the follow 12 months.

## 3 Discussion

The distinction between type 1 and type 2 diabetes mellitus is usually straightforward.^[7]^ However, as reported in this case, there may be an overlap in the presentations of the 2 disorders, which creates a diagnostic dilemma.^[8–10]^ Questions raised as we review the clinic process of the young ketosis prone diabetes patients. How to classify type of diabetes for the young patient based on his initial presentation? What was the likely reason for his metabolic decompensation? The young patient presented with marked dehydration and uncontrolled hyperglycemia in the absence of any precipitating factor such as infection., along with diabetic acidosis and ketosis resulting from deficient insulin secretion, However, the phenotypic features of obesity, acanthosis nigricans, marked insulin resistance and strong family history of adult onset diabetes which are classically associated with classic T2DM.^[11,12]^ All findings were consistent with atypical diabetes or ketosis-prone diabetes, which first systematically reported by Winters and his colleges in 1987.^[5]^ KPDM is an emerging heterogeneous syndrome.^[6,9,13–15]^This syndrome of episodic diabetic ketoacidosis without immunologic markers of type 1 diabetes is characterized by insulin dependence at the time of presentation, but followed by absence of insulin requirements for years as observed in type 2 diabetes.^[16]^ Because of the mixed features of type 1 and type 2 diabetes, this variant of diabetes has been referred to in the literature as diabetes type 1B, idiopathic type 1 diabetes, atypical diabetes, Flatbush diabetes, and more recently, ketosis-prone type 2 diabetes.^[17,18]^

The pathogenesis of this syndrome is unclear, even though the environmental factors, such as diet and physical activity, coupled with still largely unknown genetics factors clearly interact to produce the syndrome.^[19]^ What causes the initial beta-cell insult leading to acute insulin deficiency? Glucose toxicity has been suggested as a contributing factor. Accumulating data suggest that severe glucotoxic blunting of an intracellular pathway leading to insulin secretion may contribute to the reversible beta cell dysfunction characteristic of KPDM patients.^[20]^ One prospective study of patients presenting with DKA demonstrated a lower glucagon-mediated C-peptide response in obese patients with diabetic ketoacidosis compared with ketosis-resistant hyperglycemia patients, suggesting that there may be other causes and mechanisms involved. Prior studies by our group and other investigators indicate that most patients with ketosis-prone type 2 diabetes had a higher prevalence of a parental history of diabetes and were generally more obese.^[13,15]^ Because of its association with obesity and hyperlipidemia as well as hyperglycemia, some investigators have examined whether high levels of free fatty acids or other lipids might be the trigger of unproved diabetic ketosis.^[20]^ Is it possible that the ketosis-prone diabetes in Chinese patients represents an attack by disease sources factor such virus or bacteria? However, there is no evidence that those patients had an infectious illness.^[7]^ Why are Asian more susceptible to glucose toxicity and lipotoxicity? Differences in the lifestyle, including the consumption of rice and wheat as staple food or gene-environment interactions may also have an impact on the incidence of KPDM and the findings call for further studies.^[21]^

What is the best clinic treatment strategy and option? All patients with KPDM should be treated according to established principles of acute management of metabolic decompensation. Insulin replacement therapy is necessary at the acute state of insulin deficient and hyperglycemia crisis. Lifestyle changes, including optimal diet, weight loss, exercise and smoking cessation, are an important part of the treatment regimen to prevent obesity and improve insulin sensitivity, as well as to maintain good glycemic control.^[20,21]^ Screening and treatment for microvascular and macrovascular complications of diabetes should be advised according to American Diabetes Association (ADA) recommendation and long term management guidelines.^[22]^ In summary, the description is of a case of ketosis prone diabetes in an obese young Chinese teenager, who displayed uncommon clinic presentation and findings typical of both type 1 and type 2 diabetes. However, after initial insulinization near-normoglycaemic control and restoration of normotriglycemia rapidly lead to improvement in beta-cell function. The patient was able to come off insulin therapy without relapse of ketoacidosis as previous reported. His unprovoked DKA, negative auto-antibodies and partially preserved beta cell functional reserve after the acute of diabetic ketosis suggested that he has the phenotype of “A–β^+^” KPD.

## 4 Conclusion

It is time to revisit and revises the current classification of diabetes anyway. Continued collection of data and pathophysiology analysis will further clarify the involvement of ketosis-prone diabetes mellitus and insight into this uncommon clinical presentation of diabetes.

## Acknowledgments

The authors acknowledge the invaluable collaborative efforts of the staff of the Emergency Department, and Division of Endocrinology and Metabolism. The authors would like to thank our patient and his parents for all their help and enthusiasm.

## Author contributions

**Conceptualization:** Huiwen Tan, Yerong Yu.

**Data curation:** Huiwen Tan, Chun Wang.

**Formal analysis:** Huiwen Tan.

**Investigation:** Chun Wang.

**Methodology:** Huiwen Tan.

**Project administration:** Yerong Yu.

**Resources:** Yerong Yu.

**Writing – original draft:** Huiwen Tan.

**Writing – review & editing:** Chun Wang, Yerong Yu.

Huiwen Tan orcid: 0000-0002-0451-8283.
